# YOLO-ODD: an improved YOLOv8s model for onion foliar disease detection

**DOI:** 10.3389/fpls.2025.1551794

**Published:** 2025-05-22

**Authors:** Anusha Raj, Mukund Dawale, Sagar Wayal, Kiran Khandagale, Indira Bhangare, Susmita Banerjee, Ashwini Gajarushi, Rajbabu Velmurugan, Maryam Shojaei Baghini, Suresh Gawande

**Affiliations:** ^1^ Indian Council of Agricultural Research (ICAR)-Directorate of Onion and Garlic Research, Pune, India; ^2^ TIH Foundation for Technology Innovation Hub, Internet of Things and Internet of Everything (IoT & IoE), Mumbai, India; ^3^ Indian Institute of Technology, Bombay, India

**Keywords:** artificial intelligence, disease detection, deep learning, YOLOv8, image annotation, onion

## Abstract

Onion crops are affected by many diseases at different stages of growth, resulting in significant yield loss. The early detection of diseases helps in the timely incorporation of management practices, thereby reducing yield losses. However, the manual identification of plant diseases requires considerable effort and is prone to mistakes. Thus, adopting cutting-edge technologies such as machine learning (ML) and deep learning (DL) can help overcome these difficulties by enabling the early detection of plant diseases. This study presents a cross layer integration of YOLOv8 architecture for detection of onion leaf diseases *viz*.anthracnose, Stemphylium blight, purple blotch (PB), and Twister disease. The experimental results demonstrate that customized YOLOv8 model YOLO-ODD integrated with CABM and DTAH attentions outperform YOLOv5 and YOLO v8 base models in most disease categories, particularly in detecting Anthracnose, Purple Blotch, and Twister disease. Proposed YOLOv8 model achieved the highest overall 77.30% accuracy, 81.50% precession and Recall of 72.10% and thus YOLOv8-based deep learning approach will detect and classify major onion foliar diseases while optimizing for accuracy, real-time application, and adaptability in diverse field conditions.

## 1 Introduction

Onion (*Allium cepa* L.) is an important vegetable crop consumed. Onion crop suffer from multiple diseases throughout its life cycle. The incidence of various diseases significantly affects the yield and quality of onion crop. Pests and diseases collectively cause 30-50% bulb yield losses (Larentzaki et al., 2007; Nault and Shelton, 2010). Timely and accurate identification of plant diseases is of great significance for protecting crop safety and controlling the spread of diseases (Iqbal et al., 2018). The present method of disease identification is based on visual inspection of symptoms by plant protection experts. The accuracy of this identification is based on the experience and skill levels of the expert. To increase the accuracy of disease detection, several researchers have built disease detection models to classify and identify plant diseases using image processing techniques, machine learning, and deep learning algorithms.

Previous reports on plant disease detection in various cropping scenarios are briefed here, Fuentes et al. (2017) used “deep learning meta-architectures” to identify nine different kinds of pests and diseases in tomato plants using pictures taken in camera at different resolutions. In another study, Cassava leaf diseases were detected using a Convolutional Neural Networks (CNN) model, trained with field image dataset (Sambasivam and Opiyo, 2021). Pre-trained transfer learning techniques such as AlexNet and GoogleNet were applied to identify soybean diseases (Jadhav et al., 2021). The CNN model was created to classify purple blotch disease in onion crop by employing a pre-trained InceptionV3 model (Zaki et al., 2021) A deep neural network model proposed to automatically detect onion downy mildew using pictures taken periodically by a field-monitoring system of onion fields (Kim et al., 2020).

When evaluated on the tomato leaf diseases dataset, the YOLOv5 model exhibited an impressive accuracy rate of 93% (Rajamohanan and Latha, 2023). YOLOv8 has been used to increase the efficiency and precision of rust disease classification in fava bean fields (Slimani et al., 2023). The application of the YOLO model, specifically YOLOv8 and YOLOv9, for the identification of plant diseases in a hydroponic setting, was examined. The findings demonstrated that YOLOv9 outperformed YOLOv8 by a small margin in terms of detection accuracy, with 88.38% and 87.22%, respectively. For real-time plant disease detection, YOLOv8 uses less time and processing resources than YOLOv9 (Tripathi et al., 2024). A combination of YOLOv5 and YOLOv8 models was more effective for the same diseases, achieving a detection accuracy between 86.6% and 94.3%, according to Ahmed and Abd-Elkawy (2024). Further, the diseases tomato splitting, sun-scaled rot, and blossom end rot were identified with a 93.6% accuracy using YOLOv8 and YOLOv9 models, as noted by Zayani et al. (2024). Blossom end rot, splitting, and sun-scaled rot were detected using the YOLOv8 model with an accuracy of 66.67%, as reported by Iren (2024).

Deep Learning (DL) has opened new avenues for automatic plant disease identification through object detection and image classification. There are two major categories of object detection models which differs in architecture, time needed, dataset needed and accuracy. 1) Two stage Models- Fast R-CNN, Faster R-CNN, Mask R-CNN and 2) one stage includes, YOLO (You Only Look Once), Retina Net etc.

In this study, we propose cross layer integration of YOLOv8 model for improved accuracy in disease detection. YOLO is a real-time object detection method that uses a neural network to process a picture in a single forward pass. YOLO completes bounding box regression and object recognition in a single step, in contrast to conventional object detection techniques, which require several processing steps. Multiple versions of YOLO have been created, from YOLOv1 to the recently developed YOLOv11. Every new version builds upon previous versions, offering more features, including increased precision, quicker processing, and better object handling. YOLOv8 is a cutting-edge model for object recognition that evaluates photographs in the range of 40–155 frames per second (FPS) depending on the configuration. This novel method of image analysis divides an image into several grid cells and predicts the bounding box coordinates and class probabilities using a single neural network. This results in a faster and more precise disease identification process by enabling more efficient and accurate image assessment (Orchi et al., 2023). Pretrained object detection models are available in different repositories these models are trained on large datasets however training from scratch on custom dataset has added advantage of avoiding possible learning bias due to objective function difference and limited design space on networks (Shen et al., 2025).

Grad-CAM visualization technique used to highlight important regions in image which enhances model trust and accuracy. AI-driven deep learning models have been widely used for diagnosing plant diseases in major crops, including rice, wheat, maize, tomato, banana, apple, grapes, citrus, mango, tea, cucumber, cassava, ginger, sugarcane, papaya, common bean, and pearl millet. Many of these studies rely on the PlantVillage dataset, which includes only a limited selection of diseases from specific crops. However, onion diseases are not covered in the PlantVillage database. Onion pathogens and symptoms are unique, and only a few studies have explored image-based detection, typically focusing on just one or two diseases. While Jahan M. et al. (2024) conducted a comprehensive study on onion leaf disease classification and hierarchical image feature extraction, no credible image-based detection model currently exists for onion disease identification.

Currently, onion disease diagnosis depends on human expertise, with accuracy varying based on the specialist’s experience and skill level. To address this gap, we aim to develop an automated image-based object detection model for onion disease diagnosis, designed for deployment in a mobile camera-based application. This study aims to authenticate a robust onion disease detection model by analyzing the effectiveness of YOLOv5, YOLOv8, and customized YOLOv8 models using different attention modules in detecting onion diseases and evaluate their performance across different disease categories.

This paper addresses the need of a robust digital guide for realtime assistance to onion growers upon integration of this model into smartphone app (iSARATHI) (https://play.google.com/store/apps/category/FAMILY?hl=en-US). The model was optimized by integrating CBAM attention after C2f in neck and backbone network in addition dynamic task align head was replaced with detection head in YOLOv8 architecture which enhances feature extraction especially for smaller disease spot detection. The findings of this study will contribute to advancements in precision agriculture and smart disease management systems for onions.

### 1.1 Brief overview of related research

Leaf disease recognition has been extensively studied using various computer vision techniques, including traditional machine learning, deep learning-based CNNs and recent advancements in YOLO architecture. Most research efforts focus on image classification and bounding box-based detection using existing datasets. However, complex leaf diseases often have irregular shapes that bounding boxes cannot effectively capture. To address this, we integrated bounding box-based annotations utilizing real-time field data collected directly from agriculture research field plots which were then trained with customized YOLOv8, making it a more reliable and practical solution for farmers and agronomists. An illustration of previous modalities utilized for plant disease recognition enabled by object detection algorithms is described in **Table 1**.

**Table 1:** Comparative interpretation of different deep learning models previously used in object detection.

Model	Dataset	Task	Utility	Reference
YOLO-ACT (YOLOv8s)	Open-source dataset of apple leaf diseases- PlantDoc and AppleLeaf9	Disease detection	Uses cross-layer integration to improve detection accuracy	Zhang et al., 2024.
YOLOv8	Roboflow dataset (Leaf diseases)	Disease detection	GOCR EELAN module for enhanced feature extraction and WSIoU loss function to improve convergence speed and localization accuracy	Wen et al., 2025.
YOLOv8	Greenhouse vegetable disease dataset built by collecting online videos	Disease detection	Occlusion perception attention module to diminish background and HIoU loss function to address regression loss	Wang et al., 2024.
MSAM-YOLO (YOLOv8)	PlantVillage and other network datasets	Disease identification	Multiscale attention mechanism to capture subtle features of disease	Xie and Ding, 2025
TSBA-YOLO (YOLOv5)	Custom dataset on Tea leaf disease created using drone and mobile photography	Disease detection	Feature fusion network and spatial feature fusion to resist background inference	Lin et al., 2023
YOLOv8	Mixed dataset containing images collected from the field and the internet	Detection and categorization of tomato fruits	Depthwise separation convolution to reduce computational complexity and Feature enhancement module to avoid occlusion due to overlaps in the images	Yang et al., 2023.
YOLOv8n	Open source apple leaf disease dataset -AppleLeaf9	Disease detection	C3 Ghost convolution to reduce parameter count and a global attention mechanism with a bidirectional feature pyramid network for small lesion detection	Gao et al., 2024.
Pyramid-YOLOv8 (YOLOv8x)	Rice blast disease dataset created using smartphone	Disease detection	Multi attention feature fusion network to enhance model precision	Cao et al., 2024
CAM-YOLO YOLOv5)	Lobaro tomato dataset	Detection and classification	Convolutional block attention module and Distance oversection over union to detect overlapped and small objects	Appe et al., 2023
YOLOv8	Sourced from Kaggle	Classification	Anchor boxes were added to align with size and shape of disease patterns	Ghafar et al., 2024
EggplantDet (YOLOv8)	Custom dataset consisting images collected by inspection robots and smartphones	Disease detection	FasterNet for efficient feature extraction and Tripple Attention Mechanism for small object detection	Liu and Wang, 2025
Alpha-ElOU-YOLOv8 (YOLOv8n)	Custom dataset of rice leaf disease images	Disease detection	Uses EloU and alpha loss function instead of box loss function to enhance model performance	Trinh et al., 2024
YOLOv8	Custom dataset of tomato leaf disease images	Disease detection	Uses Grouped Depthwise Convolutions and Squeeze and Excitation blocks to improve model accuracy	Shen et al., 2025

This table explains both parallels and contrasts among a subset of previously built plant disease detection models. This manifests the importance of this research work completed using a dataset generated in natural settings.

## 2 Materials and methods

### 2.1 Study area

The photographs were captured at ICAR-Directorate of Onion and Garlic Research (DOGR), Pune, Maharashtra, India, centered at latitude 18°50’27.99”N, longitude 73°53’12.88”E EPSG:4326 WGS 84/UTM zone 43°N over the span of 4 months from September to December 2023.

### 2.2 Image collection

**Figure 1:**
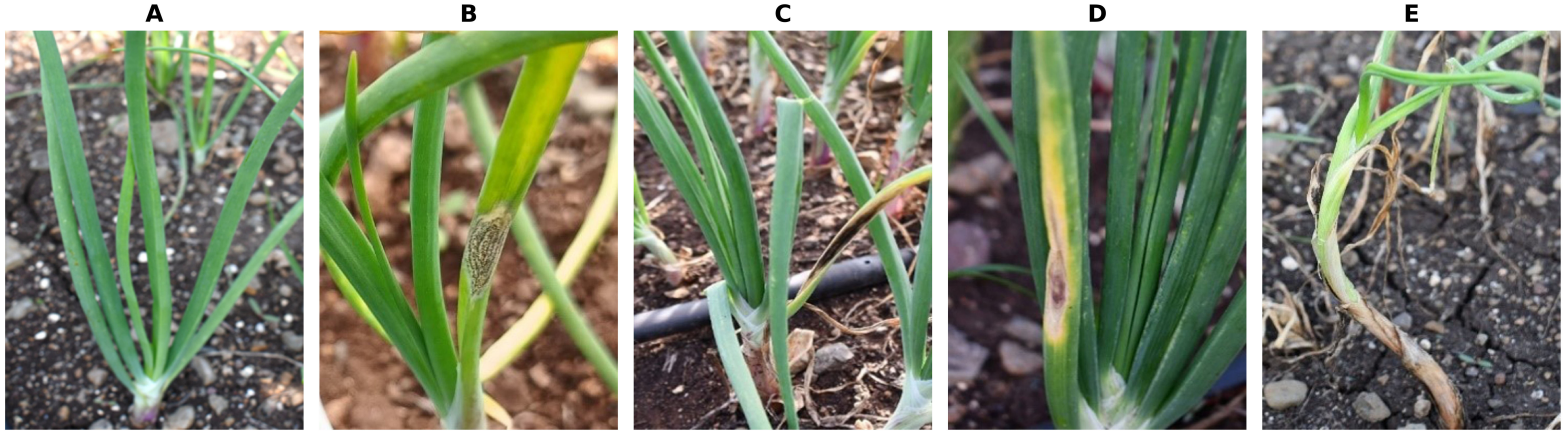
Images of diseased and healthy onion plants **(A)** Healthy plant **(B)** Anthracnose **(C)** Stemphylium **(D)** Purple blotch **(E)** Twister disease.

The photographs were taken in a natural environment using a Nikon D7500 DSLR camera with 4000 x 6000 pixels resolution and a Moto g72 smartphone with an image resolution of 3000 x 4000 pixels. The camera was positioned 0.3 to 0.5 m away from the plants. Images of both healthy and diseased plants were obtained. Onion plants were photographed in multiple orientations so that the main features of the disease, such as texture, coloration, shape and morphology depended on the extent of the damage. Four major diseases of onion, such as Anthracnose, Twister disease, Stempylium blight and Purple blotch were the focus of model development. **Figure 1** depicts images of the above-mentioned diseases taken from the symptomatic onion plants.

### 2.3 Data preprocessing

**Figure 2:**
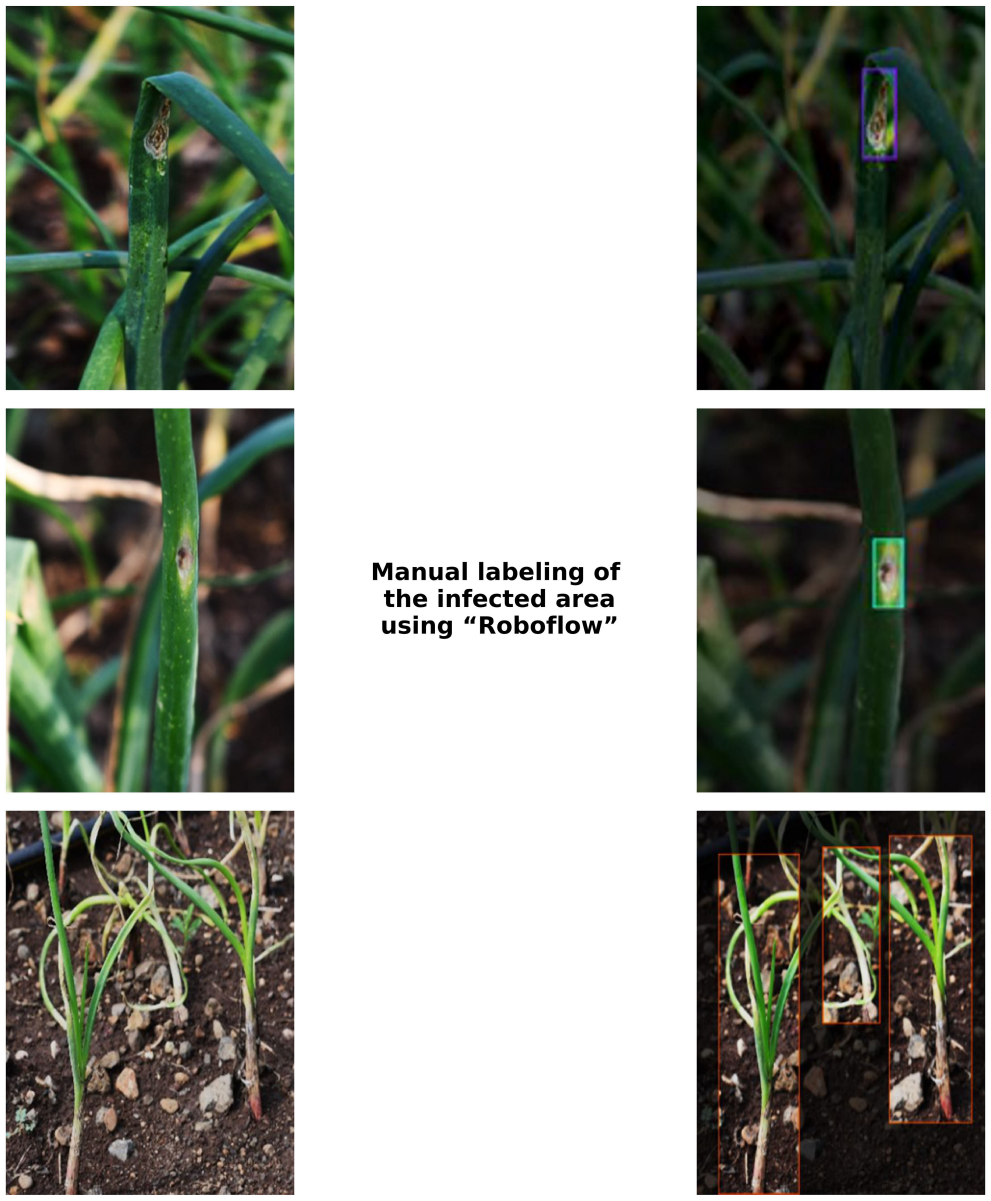
Annotated images.

From the captured images, 1000 images were chosen to generate a dataset for this study. Among the 1000 images, 800 images were equal number of diseased plants infected by Anthracnose, Stemphylium blight, Purple blotch and Twister disease; and the remaining 200 images were from healthy onion plants. A computer vision platform ‘Roboflow’ was used to manually label the disease symptoms. Roboflow supports various annotation formats (bounding boxes, polygons, masks, keypoints) for diverse computer vision tasks (https://roboflow.com/formats). Real-time collaboration ensures consistent annotations, while pre-labeling with existing models greatly reduce human effort. The images were uploaded batch-wise and image annotation was done using such as bounding box feature. **Figure 2** shows the manually annotated images that were resized to a resolution of 640 x 640. Data augmentation techniques, such as flipping, rotation and scaling, were utilized to enhance the dataset and mitigate overfitting. The images were augmented with a 50% probability of horizontal flip, and salt-and-pepper noise was applied to 0.1% of the pixels. After augmentation 1391 images were used for training the model, 189 for validation and 100 for test.

### 2.4 Model training and architecture of YOLO

**Figure 3:**
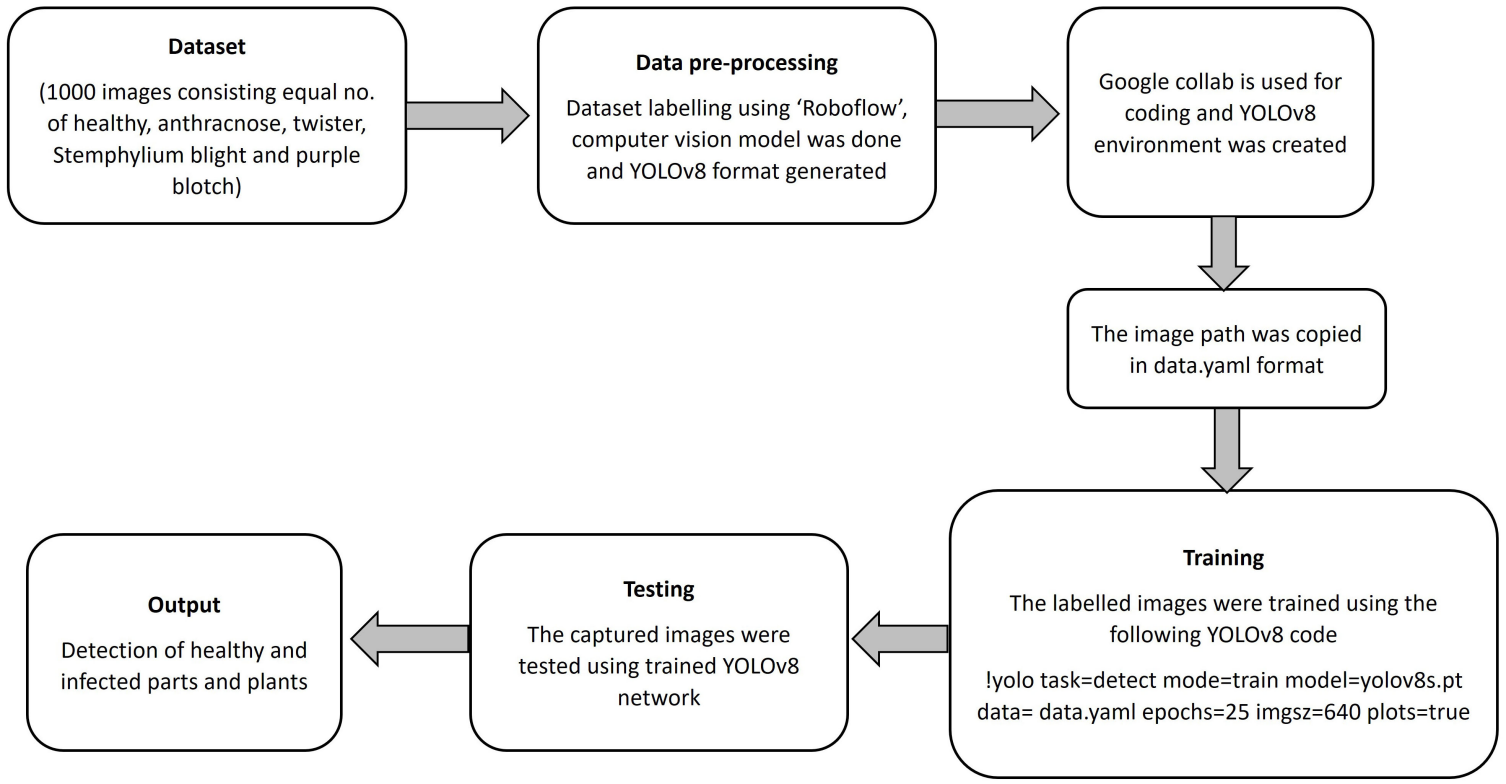
Block diagram of the complete flow of YOLOv8 model trained and tested on our dataset.

**Figure 4:**
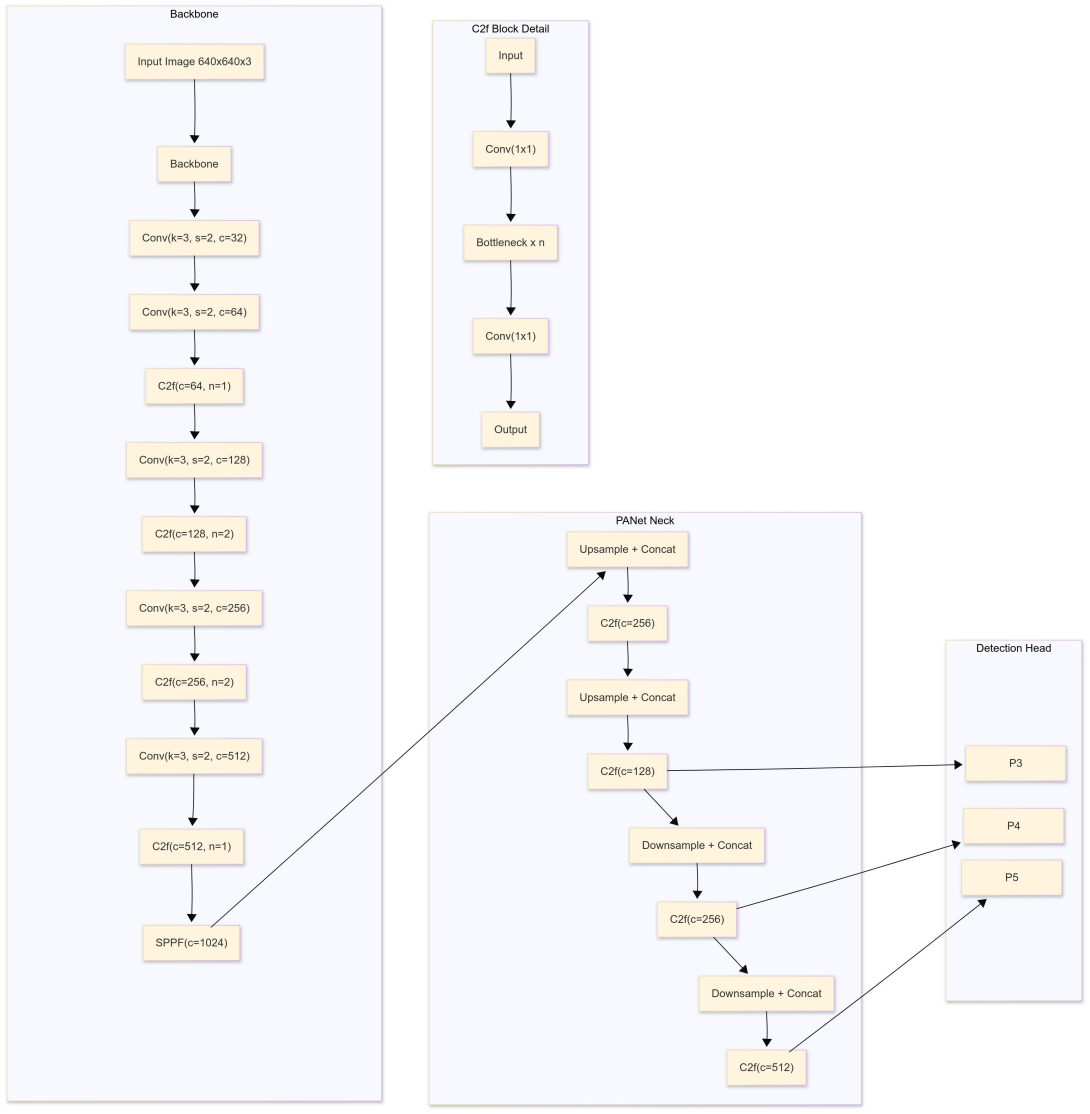
Schematic diagram of yolov8s model.

The YOLOv8 and YOLOv5 network are similar, consisting mainly of the backbone, neck and head. YOLO is an object detection algorithm that can identify and detect objects in an image. By applying the selected YOLO models to the preprocessed dataset, these YOLO models will train the data to detect object. YOLO models were employed for training because of its advanced capabilities for real-time object detection and superior performance in terms of speed and accuracy. YOLOv8 is a state-of-the-art object detection, segmentation, and classification algorithm developed by Ultralytics. It builds on previous YOLO versions with improved accuracy, speed, and flexibility. Key features include anchor-free detection, a dynamic backbone, and a unified framework for multiple vision tasks. YOLOv8 isoptimized for real-time applications and supports deployment on edge devices. The training process involved the data pre-processing methods and the data.yaml file, a configuration file integral to the YOLOv8 framework, which is programmatically generated to encapsulate essential metadata about the dataset, including class labels, directory paths for training, validation, and testing datasets, as well as the number of instances per class. The file served as a critical input for the YOLOv8 pipeline, enabling streamlined preprocessing and facilitating efficient integration of the model with the dataset during the training process. The models were fine-tuned over 50 epochs with a batch size of 16, utilizing the Adam optimizer and a learning rate scheduler for optimal convergence. Here, Adam optimizer was used since it enhances YOLOv8 for onion disease detection by handling small, variable symptoms like browning and lesions. Its adaptive learning rates help the model adjust to image variations, while its robustness ensures reliable training even with noisy or incomplete data. Adam’s fast convergence makes it ideal for real-time disease detection, enabling timely agricultural decisions (Ghafar et al., 2024). **Figure 3** shows a block diagram of the training and testing frameworks of the proposed model. The architecture of a basic object detection model consists of a backbone, a neck and a head (**Figure 4**). Meaningful feature extraction from the input is the responsibility of the backbone, which is also referred to as a feature extractor. The neck operates as a bridge between the backbone and the head, executing feature fusion processes and integrating contextual information. The head was responsible for producing outputs, bounding boxes, class predictions, and confidence scores for detected objects. The primary features of YOLOv5 and YOLOv8 include anchor-free detection, mosaic data augmentation, C2f module, decoupled head, and a modified loss function (Sandhya and Kashyap, 2024). Mathematical equations of YOLOv8 are given below:

1. Object detection problem formulation:

Object detection is treated as a regression problem, where the goal is to predict both the class of an object and its bounding box from a given input image. Each bounding box represented by four coordinates


    x, y−coordinates of center of bounding box

w, h−width and height of bounding box, relative the entire image  

Class prediction: for each bounding box YOLO predict the probability distribution over the class labels.

C – no of classes,
S – no of cell grid in image,
B – no of bounding boxes per cell.

2. YOLOv8 Architecture:

Backbone: The backbone extracts features from the input image using a convolutional neural network (CNN). YOLOv8 employs the CSPDarknet architecture with ELAN (Efficient Layer Aggregation Networks), which enhances gradient flow and efficiency


(1) F=Backbone(I)

Where F is a feature map, and I is the input image (Equation 1).

Neck: The neck helps in multi-scale feature aggregation and uses Path Aggregation Network (PAN) which combines features from different scales for better object detection across various sizes.


(2) F_neck=Neck(F)

Where *F_neck* is the feature map passed to the detection head (Equation 2).

Detection Head: The head predicts object bounding boxes, class scores, and confidence scores using anchor-free detection approach. YOLOv8 divides the image into a grid and predicts bounding boxes and their corresponding class probabilities for each cell.

The output of each cell is vector $\left[x,y,w,h,p_{1},p_{2},p_{3},\ldots p_{c}\right],$ Where, x,y,w,h are the bounding box coordinates, $p_{i}$ is the class probability for class i, C is the number of object classes.

3. Mathematical formulation for YOLOv8

Both YOLOv5 and YOLOv8 follows the regression approach to predict bounding boxes and class probabilities. The loss function use to training is a combination of several components:

Bonding Box Loss: The difference between the predicted and ground truth bounding box coordinates is measure using the IoU (intersection over Union) loss, typically combined with a smooth L1 loss.

$b_{pred}=\left(x,y,w,h\right)$ predicted bounding box and

$b_{gt}=(x_{gt},\;y_{gt},\;w_{gt},\;h_{gt})$ corresponding ground truth.

The loss can be written as: during network training, the loss function is a tool used to represent the difference between predicted and actual values. It plays crucial role in training of disease detection models. In YOLOv5s and YOLOv8s multiple loss functions are combined for training bounding box regression, classification, and confidence. The loss function use are as follows:


(3) $$\begin{array}{c} Loss_{box}=\;\lambda_{coord}\sum_{i}I_{obj}[{\left(x_{i}-x_{i,gt}\right)}^{2}+{\left(y_{i}-y_{i,gt}\right)}^{2}]\\ +\;\lambda_{wh}\sum_{i}I_{obj}[{\left(w_{i}-w_{i,gt}\right)}^{2}+{\left(h_{i}-h_{i,gt}\right)}^{2}] \end{array}$$

Where, $I_{obj}$ is the indicator function (equal to 1 if an object is present, and 0 otherwise), and $\lambda_{coord}$ , $\lambda_{wh}$ are the weights that balance different components of the loss (Equation 3).

Classification Loss: YOLOv8 also predict the class probabilities for each bounding box. The loss for class prediction is computed using binary cross-entropy


(4) $$Loss_{cls}=\;-\sum_{c}y_{cls}\text{log}\left(p_{cls}\right)$$

where $y_{cls}$ is the true class label and $p_{cls}$ is the predicted probability for the class (Equation 4).

As shown in Equation 5, the total loss is a combination of classification and box regression losses.


(5) $$Loss_{conf}=Loss_{cls}+Loss_{box}$$

4. CIoU (intersection over Union):

The CIoU is a crucial part of the bounding box prediction, and it measures the overlap between predicted and ground truth boxes. Its used to determine whether a predicted bounding box is considered a correct detection or not.


(6) $$CIoU=\;1-\text{IoU}+\;\frac{\rho^{2}\left(b_{center},b_{center}^{gt}\right)}{C^{2}}+\alpha .\upsilon$$

Where represents the intersection over Union, is the distance between the center point of the predicted box and the ground truth box, c is the diagonal length of the smallest enclosing box covering both the predicted and ground truth box, υ is the aspect ratio consistency term and α is the probability coefficient (Equation 6).


(7) $$dfl=\;-\sum_{i=1}^{n}w_{i}\text{log}\left(p_{i}\right)$$

Where $w_{i}$ is the weight usually adjusted according to the position of the true bounding box. $p_{i}$ is the probability of each class in the predicted probability distribution (Equation 7).

Convolutional Block Attention Module

**Figure 5:**
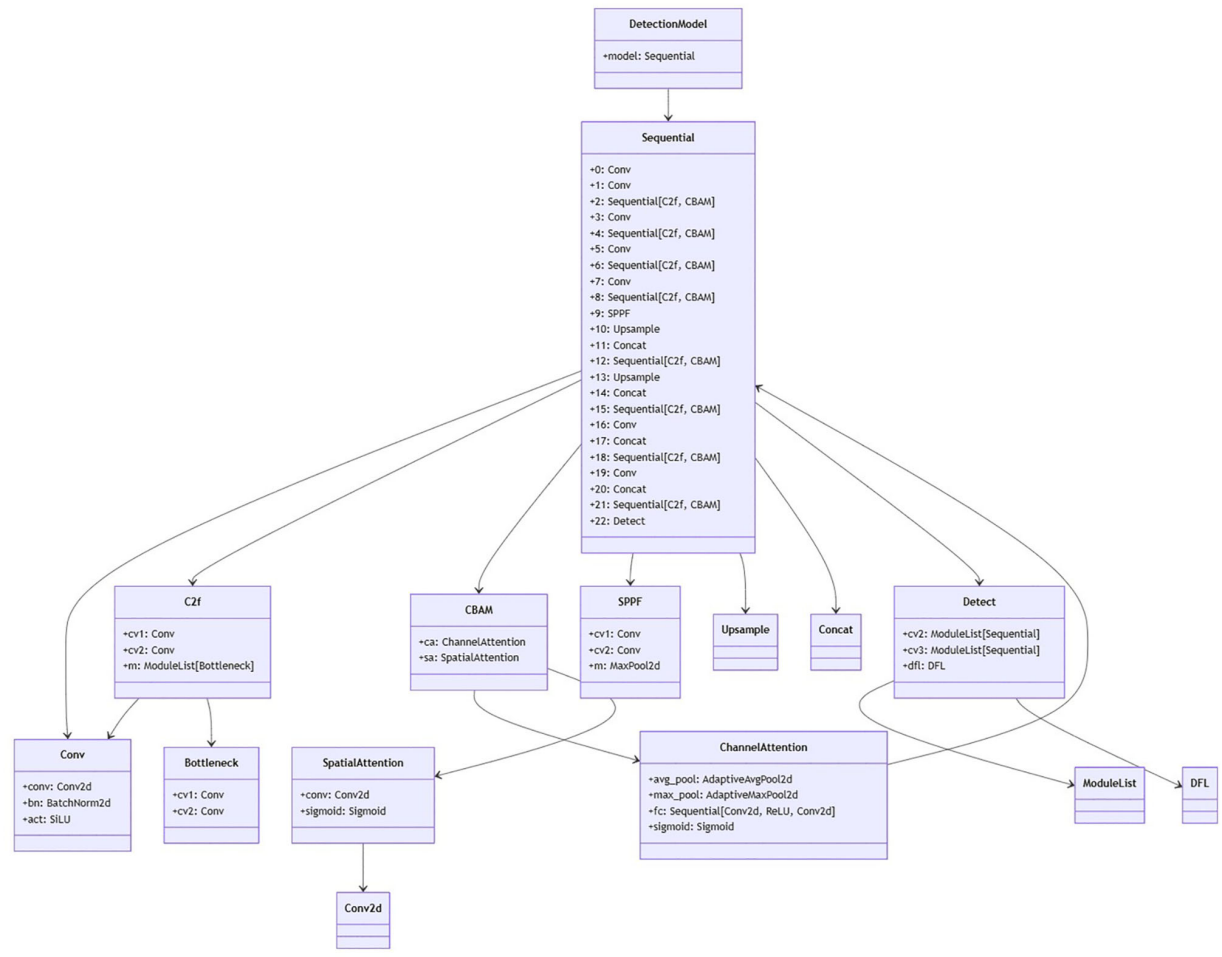
Schematic diagram of Yolov8+CBAM.

Convolutional Block Attention Module (CBAM) is an attention module for convolutional neural networks. CBAM is simple yet effective attention module for feed-Forword convolutional neural networks (**Figure 5**). Given an intermediate feature map, the module sequentially infers attention maps along two separate dimensions, channel and spatial, then the attention maps are multiplied to the input feature map for adaptive feature refinement. CBAM combine channel and spatial attention mechanism, effectively identified the key features in the images while suppressing irrelevant noise. This dual attention mechanism notably enhances the accuracy and efficiency of detection (Woo et al., 2018).

Channel Attention (CAM) identifies “what” features are important by analyzing channel-wise relationships. It applies both average and max pooling to capture global context, processes them through a shared MLP with bottleneck reduction (e.g., reducing channels by ratio r=16), and generates a channel attention mask via sigmoid activation. This mask highlights informative channels while suppressing less relevant ones.

Average Pooling:


(8) $$F_{avg}^{c}=\frac{1}{H\;\times W}\sum_{i=1}^{H}\sum_{j=1}^{W}F^{c}\left(i,j\right)$$

Output: F_avg (Equation 8)

Max Pooling as shown in Equation 9:


(9) $$F_{max}^{c}=maxF^{c}\left(i,j\right)$$

Shared MLP(Bottelneck): with reduction ratio r:


(10) $$MLP(F_{pool)}=\;W_{1}\;\left(ReLU\left(W_{0}F_{pool}\right)\right)$$

Where: $W_{0}\in \;{\mathbb{R}}^{\left(C/r\right)\times C}$ weight for dimension reduction,

$W_{0}\in \;{\mathbb{R}}^{\left(C/r\right)\times C}$ weight for dimension restoration (Equation 10).

Combine and Activate:

Sum the MLP outputs and apply sigmoid( σ ) to generate the channel attention mask M_c (Equation 11):


(11) $$M_{c}\left(F\right)=\;\sigma \;(MLP\left(F_{avg}\right)+\;(MLP\left(F_{max}\right)$$

Apply to input features:


(12) $$F^{\prime }=M_{c}\;\left(F\right)\;⊗F$$

Where: denote ⊗ the element wise multiplication (Equation 12).

Average pooling capture global context, while max pooling preservers salient features combine both improves robustness. Bottleneck (r=16) balances efficiency and effectiveness by reducing MLP parameters. Sigmoid normalizes the attention weights to [0,1], acting as a soft feature selection.

Spatial Attention module (SAM) Formulas: spatial attention module is comprised of a three-fold sequential operation. The first part of it is called the channel pool, where the input tensor of dimensions (C×H×W) is decomposed to 2 channels, i.e. (2×H×W), where each of the two channels represents max pooling and average pooling across the channels. This serve as the input to the convolutional layer which output a 1-channel feature map, i.e. the dimension of output is (1×H×W).

Given the channel-refined feature map F’ ∈ ℝ^(C×H×W) from CAM: the spatial attention mechanism follows these steps

Compute average-pooled features across channels as shown in Equation 13:


(13) $$F_{avg}^{'}=\;AvgPool_{channel}\left(F'\right)\in {\mathbb{R}}^{1\times H\times W}$$

Equation 14 shows, where each spatial position (i,j) is the mean of all channels


(14) $$F_{avg}^{'}\left(i,j\right)=\;\frac{1}{C}\;\sum_{c=1}^{c}F_{c,i,j}^{'}$$

Equation 15 shows compute max-pooled features across channels:


(15) $$F_{max}^{'}=\;maxPool_{channel}\left(F'\right)\in {\mathbb{R}}^{1\times H\times W}$$

Where each spatial position (i,j) is the maximum across channels (Equation 16)


(16) $$F_{max}^{'}\left(i,j\right)=max_{c=1}^{C}\;F_{c,i,j}^{'}$$

Concatenate pooled features

In Equation 17 stack $F_{avg}^{'}\;$ and $F_{max}^{'}$ along the channel dimension:


(17) $$F_{concat}^{'}=Concat(F_{avg}^{'},\;F_{max}^{'})\in {\mathbb{R}}^{2\times H\times W}$$

Apply convolution to generate spatial attention pass the concatenated features through a 7×7 convolutional layer followed by sigmoid activation


(18) $$M_{s}\left(F^{\prime }\right)=\;\sigma \;\left(f_{7\times 7}\left(F_{concat}^{'}\right)\right)\in {\mathbb{R}}^{1\times H\times W}$$

In Equation 18 Where: $f_{7\times 7}$ is a convolution with single output channel, σ  is the sigmoid function, normalizing attention weight to [0,1]

Apply attention to the refined feature map multiply the spatial attention map $\;M_{s}\;$ with channel-refined feature map $F^{\prime }$


(19) $${F^{\prime }}^{'}=M_{s}\left(F^{\prime }\right)\;⊗F'$$

Where denote ⊗ the element wise multiplication (Equation 19).

Dynamic Task-Aligned Head (DTAH)

The Dynamic Task-Aligned Head (DTAH) is designed to address the inherent misalignment between classification and localization task in object detection (Feng et. al., 2021). Traditional decoupled heads process these tasks in parallel, leading to discrepancies in feature learning where classification focuses on discriminative local features, while localization requires global spatial context for precise bounding box regression. In domain disease detection, the accuracy of both task is indispensable. DTAH mitigates this issue through three key innovation:

1. Task interaction module: A shared feature extractor using grouped convolutions to explicitly model interactions between classification and localization tasks. This module generates task aware features by using multi-level spatial and semantic information, ensuring that both tasks operate on aligned feature representations.


(20) $$F_{interact}=\;\sigma \;\left(W_{2}*ReLU\left(W_{1}*F_{in}\right)\right)⊙F_{in}$$

Where in Equation 20:

$F_{in}\in {\mathbb{R}}^{H\times W\times C}$ Input backbone features.

$W_{1,}W_{2}$ : Grouped convolutional kernels

σ : Sigmoid activation for attention gating.

⊙ : Element-wise multiplication.

2. Deformable Localization branch: Incorporates deformable convolutional (DCN) in the localization pathway to dynamically adjust receptive fields based on object geometry. This allows the model to adapt to irregular disease pattern by predicting per pixel sampling offsets, enhancing boundary precision.

For adaptive spatial sampling, deformable convolutions predict offsets $\Delta p_{k}$ for each kernel position $p_{k}$


(21) $$F_{deform}=\sum_{k=1}^{k}w_{k}.F_{interact}\left(p+p_{k}+\text{Δ}p_{k}\right)$$

Where in Equation 21: $\Delta p_{k}$ = $W_{offset}*\;F_{interact}$ (offset prediction)

$w_{k}$ : Learnable kernel weights.

3. Unified optimization:

A joint loss function balances classification accuracy and localization precession with additional alignment loss term that panelizes spatial mismatches between task specific features.

The total loss combines task specific objectives with an alignment penalty as shown in Equation 22.


(22) $$L_{DTAH}=\;\lambda_{1}L_{cls}+\;\lambda_{2}L_{reg}+\;\lambda_{3\;}\left|\left|M_{cls}-\;M_{reg}\right|\right|2\;$$

Where:

$L_{cls}$ : Focal loss classification.

$L_{reg}$ : Distribution Focal Loss (DFL) for bounding box regression.

$M_{cls}$ , $M_{reg}\;:$ spatial attention maps from each task.

$\lambda_{i}$ : Balancing weights.

**Figure 6:**
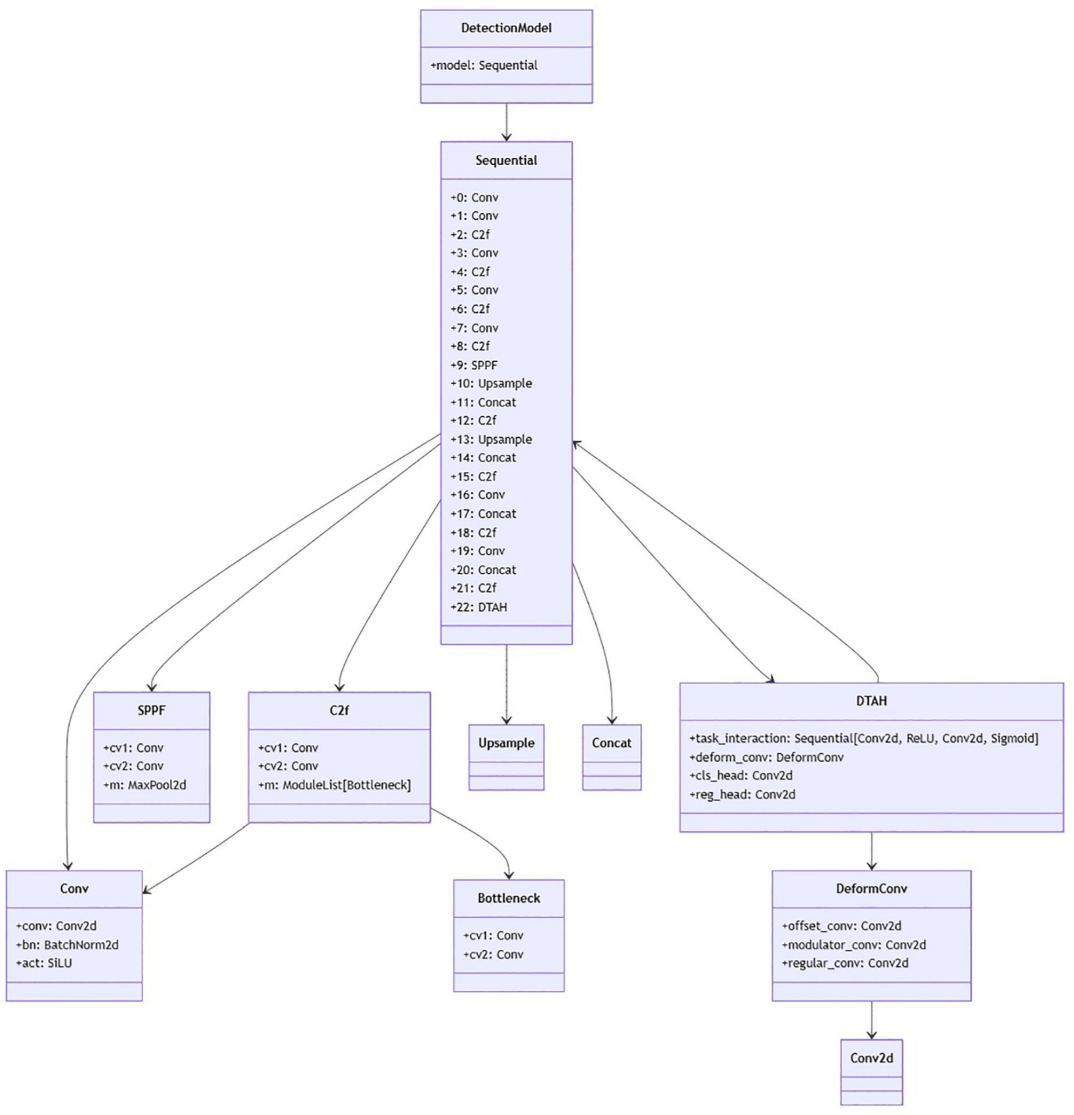
Schematic diagram of yolov8+DTAH.

The DTAH architecture is implemented as a lightweight yet powerful replacement for conventional decoupled heads in **Figure 6**. **Supplementary Figure S1** is visualization of overall architecture of proposed onion foliar disease detection model based on yolov8 integrated with CBAM (convolutional block attention module) and DTAH (Dynamic Task-Aligned Head). The diagram shows CBAM is embedded within the C2f blocks for enhanced feature extraction, and DTAH is added at the end of improve detection accuracy with deformable convolutional.

### 2.5 Model evaluation parameters

F1 Score: To evaluate the detection performance, metrics like precision, recall, and F1 score are used. For each class:

Precision: The fraction of true positive prediction among all positive predictions (Equation 23).


(23) $$Precision=\;\frac{TP}{TP+FP}$$

Recall: The fraction of true positive predictions among all actual positives (Equation 24).


(24) $$Recall=\;\frac{TP}{TP+FN}$$

F1 Score: The harmonic means of precision and recall (Equation 25):


(25) $$F1\;Score=2\;\times \;\frac{Precision\;\times Recall\;}{Precision+Recall}$$

TP (True Positive) refers to the number of correctly identified positive samples.

TN (True Negative) refer to the number of correctly identify negative samples.

FP (False positive) represent the number of negative samples incorrectly identified as positive.

FN (False Negative) refer to the number of positive sample incorrectly identified as negative.

## 3 Experimental results and analysis

### 3.1 Dataset distribution

**Figure 7:**
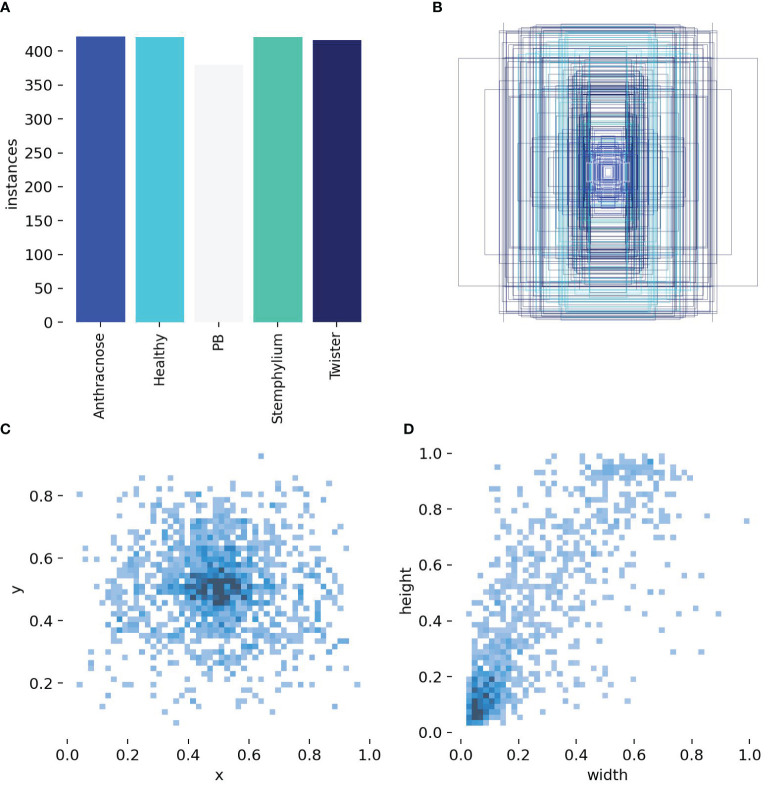
Labels and label distribution, **(A)** Bar Chart of instances per class, **(B)** Bounding box distribution, **(C)** Heatmap of bounding box centers, **(D)** Heatmap of bounding box dimensions.

The distribution of the dataset and number of instances were calculated and the results shown in **Figure 7**. **Figure 7A** shows a bar chart of instances per class. The classes represented were healthy, Anthracnose, Purple blotch (PB), Stemphylium blight and Twister disease. The training dataset is well balanced with each class having comparable number of instances Anthracnose (421), healthy (420), PB (379), Stemphylium blight (420) and twister (416) this balanced class distribution ensure that no single class dominates the dataset, reducing the risk of model bias. It enhances the model ability to generalize across different disease categories, leading to improve training and prediction accuracy. **Figure 7B** shows the bounding-box distribution. Distinct colors were used to depict boxes, which may indicate distinct classes. In some images, items were frequently found in the center, as indicated by the overlap and concentration of boxes in the center which could lead to a central bias in the model. **Figures 4C** and **7D** show a heatmap of bounding box centers (x, y) and a heatmap of bounding box dimensions (width and height) respectively. The heat map shows that the majority of the objects are situated close to the center of the image, and the majority of items in the dataset are comparatively small, as evidenced by the large concentration of bounding boxes with smaller width and height values. Uniformity in object dimensions could lead to quicker model convergence but may not generalize well to real-world data with varied object sizes. The correlogram of the custom dataset (**Supplementary Figure S2**) highlights the model’s detection confidence across image regions. This analysis could help us adjust the parameters of the object identification model, such as adjusting anchor sizes, balancing the dataset, or employing data augmentation to lessen the central bias.

### 3.2 Training and validation performance metrics

**Figure 8:**
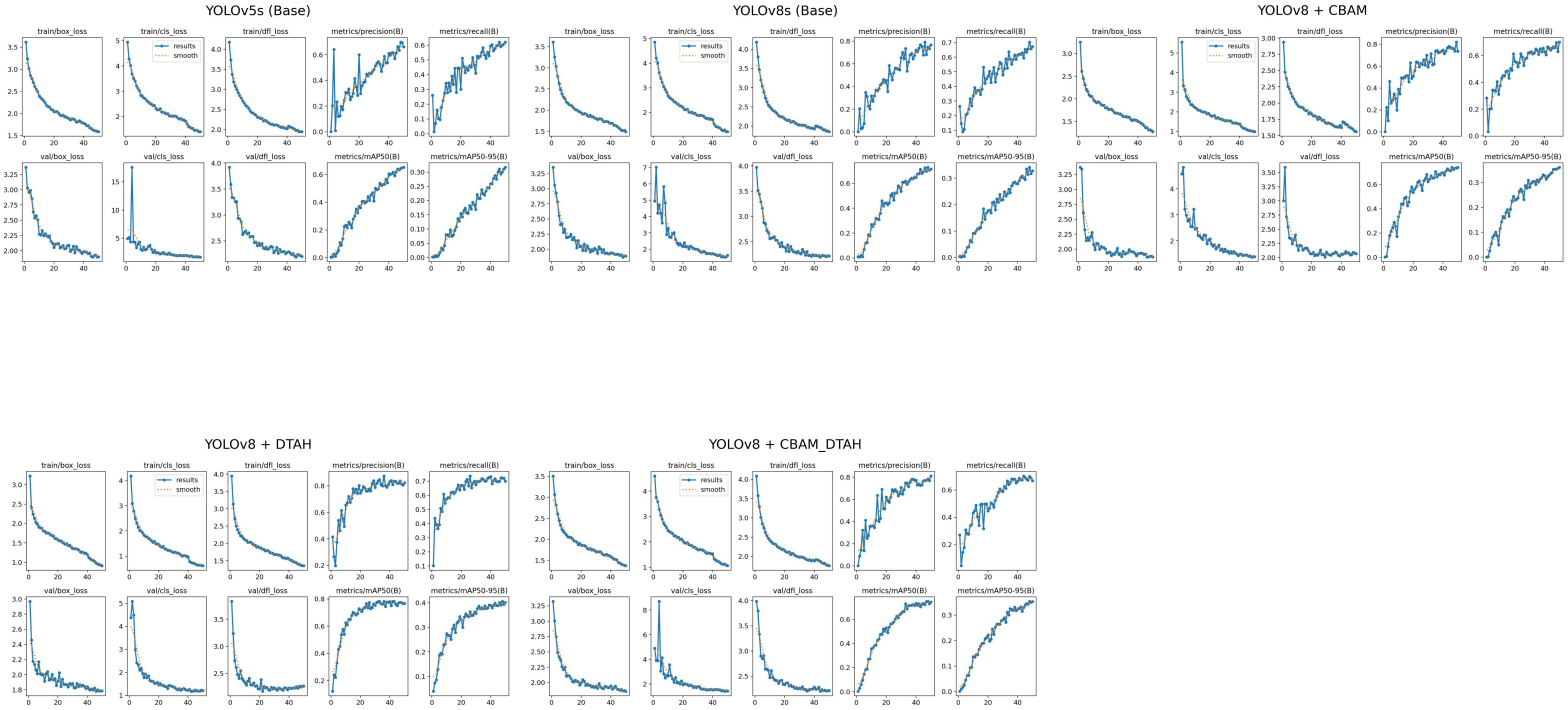
Visual analysis of model evaluation indicators (Precision, recall, and mAP@0.5) during training.

**Figure 9:**
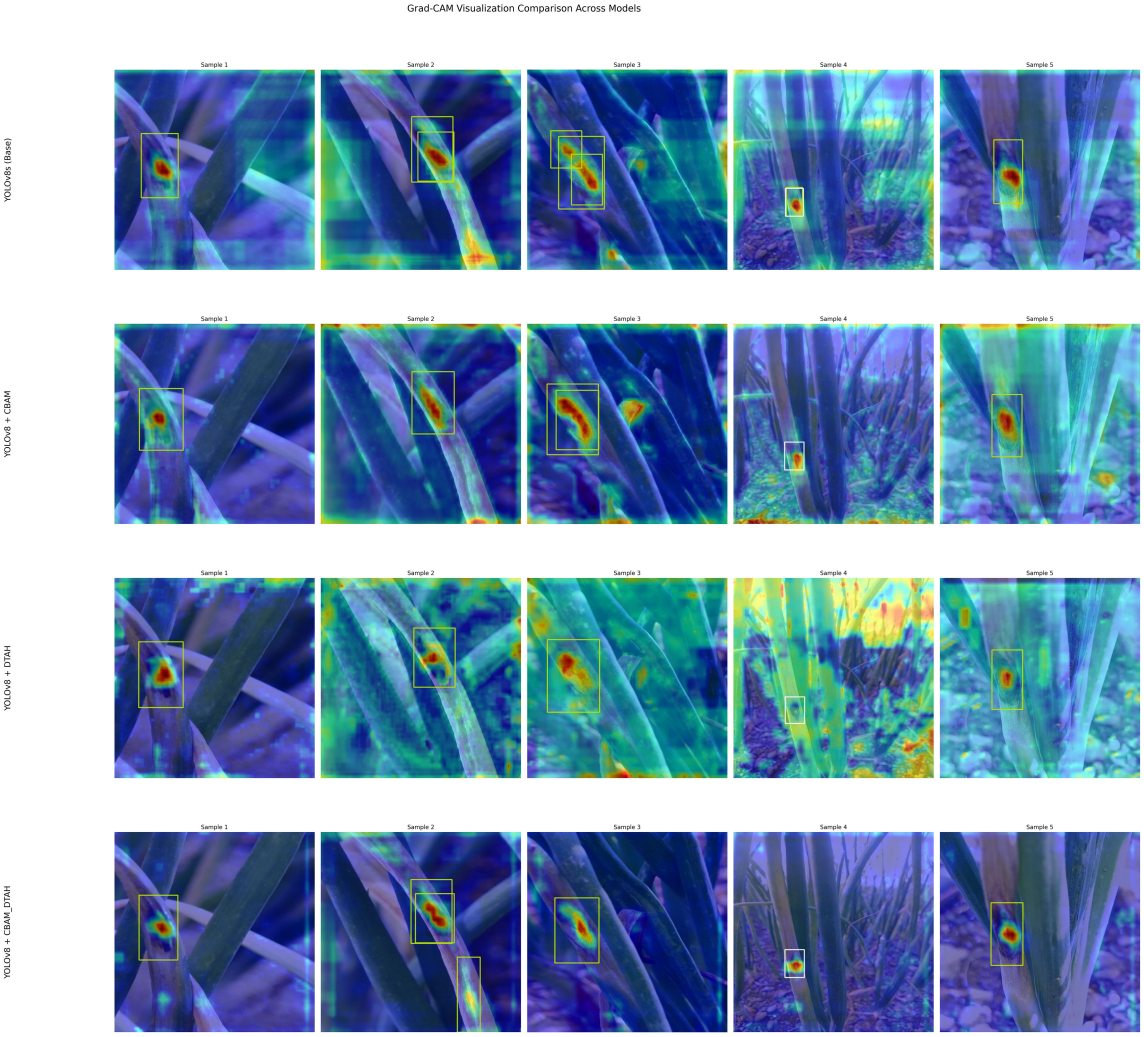
Grad CAM heatmap model wise comparison.

The performance metrics of the model are shown in **Figure 8**. With all training losses such as box, classification, and DFL (Distribution Focal Loss) gradually declining, the improved YOLOv8s model exhibits learning to predict more precise bounding box details, suggesting enhanced object localization and classification on training data. indicating that the model is generally effective on unseen data. CABM and DTAH attention mechanisms were applied to improvise overall precision and accuracy in disease detection by localization of smaller disease spots in the image. Grad cam heatmaps were adapted for comparative visualization of detection accuracy (**Figure 9**). The overall performance demonstrates that the model is capable of accurate detection while maintaining sensitivity to true positives. Overall, the model showed good convergence and encouraging outcomes.

### 3.3 Model performance measure

**Figure 10:**
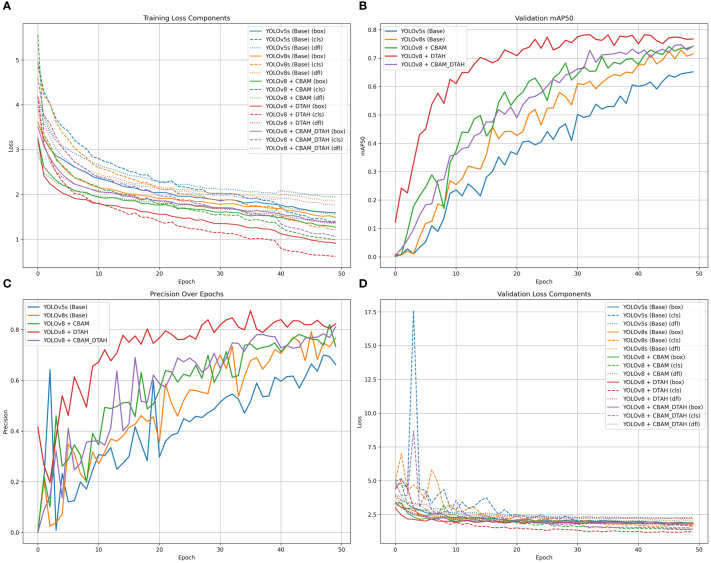
**(A)** Training loss component, **(B)** Validation mAP50 curve, **(C)** Precession over epoch curve, and **(D)**Validation loss componant.

The comparative evaluation of various YOLO architectures on the onion leaf disease detection dataset reveals significant performance variations across different model configurations (**Figure 10**). The dataset comprises five classes (Anthracnose, Healthy, PB, Stemphylium, Twister) with 189 validation images containing 257 total instances. The results demonstrate that YOLOv8 with DTAH achieved the best balance of performance metrics, attaining the highest mAP50 (0.773) while maintaining competitive inference speed (8.1ms). however base YOLOv8s model shows significant improvement over YOLOv5s (+7.5% in mAP50), and the DTAH modification provides an additional 4% boost in mAP50. Notably, the DTAH variant achieves the highest recall (0.727) among all models, indicating better detection capability for difficult cases (**Table 2**).

**Table 2:** Comparison of accuracy, precession and recall among yolo models.

Model	mAP50	Precision	Recall	Inference Speed (ms)
YOLOv5s (Base)	0.654	0.665	0.626	9.1
YOLOv8s (Base)	0.729	0.749	0.708	8.4
YOLOv8 + CABM	0.74	0.73	0.7	8.5
YOLOv8 + DTAH	0.772	0.815	0.721	8.1
YOLOv8 + CABM_DTAH	0.75	0.784	0.698	8.9

**Figure 11:**
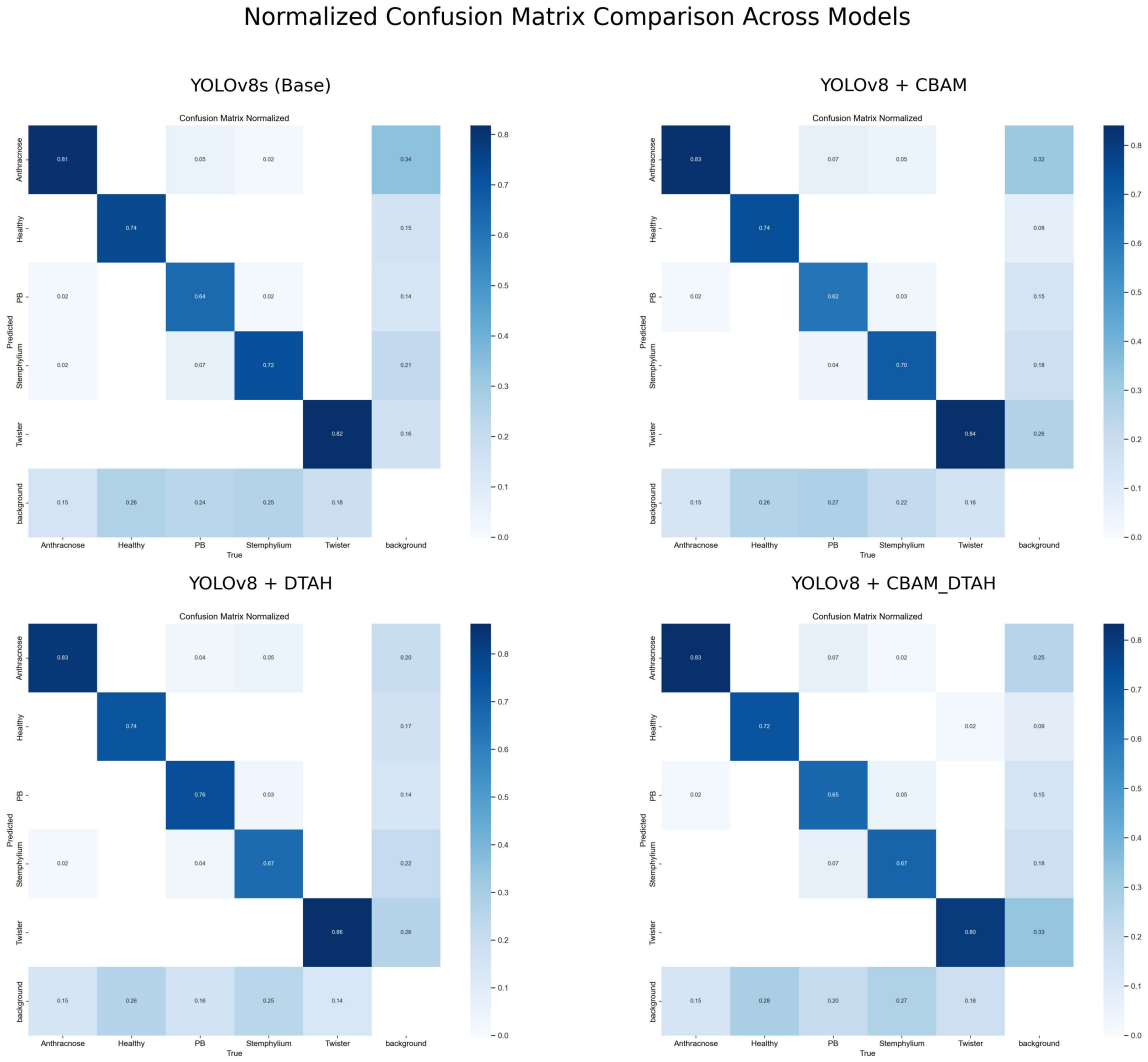
Confusion matrix diagram for the proposed YOLOv8 model.

The class-wise mAP50 performance **Table 3**, Precession recall curve (**Supplementary Figure S3**) and normalized confusion matrix (**Figure 11**) reveals several important insights about how each model variant performed across different onion leaf disease categories. Starting with Anthracnose detection, we observed a clear progression in performance from the baseline YOLOv5s (0.620 mAP50) to the enhanced YOLOv8 variants, with the DTAH modification showing particularly impressive results (0.786 mAP50) comparatively showing improvement of 17% over YOLOv5s and 9% over base YOLOv8s. This substantial boost suggests the deformable attention mechanism in DTAH is exceptionally well-suited for detecting the irregular lesion patterns characteristic of Anthracnose. The Healthy class shows more consistent performance across all models (ranging from 0.716 to 0.784 mAP50), indicating that all architectures can reliably identify healthy plants, with the CBAM+DTAH attention integrated model achieving the highest score (0.784).

**Table 3:** Comparative class wise accuracy.

Class	YOLOv5s	YOLOv8s	YOLOv8+CABM	YOLOv8+DTAH	YOLOv8+CABM_DTAH
Anthracnose	0.62	0.694	0.74	0.786	0.748
Healthy	0.716	0.741	0.764	0.768	0.784
PB	0.68	0.717	0.649	0.792	0.709
Stemphylium	0.573	0.693	0.74	0.661	0.725
Twister	0.678	0.801	0.811	0.855	0.776

For PB detection, the base YOLOv8s showed improvement over YOLOv5s (0.717 vs 0.680), with DTAH providing a modest additional gain (0.79). Interestingly, the CBAM model showed slightly reduced performance for this class, suggesting the CBAM attention may be less effective for small spot compared to disease patterns. Stemphylium detection follows a similar pattern, where CABM provides the best performance (0.742), outperforming even DTAH (0.665).

In contrast, our YOLOv8+DTAH (Dynamic class align head) modification exhibited superior performance across all metrics, achieving 77.3% mAP50 with 4.85-6.56% absolute improvement over the Detectron2 models - while using only 11.1M parameters (69% fewer than RetinaNet, 75% fewer than Faster R-CNN) **Table 4**. Remarkably, this accuracy gain comes with substantially faster inference speed (123 FPS), making it 1.9-2.8X faster than the comparative methods. The results demonstrate that the YOLOv8 architecture with DTAH attention mechanisms provides better feature representation for onion leaf disease detection compared to traditional two-stage approaches, while maintaining the computational efficiency crucial for real-world agricultural applications. The performance advantage is particularly notable given the model’s compact size, suggesting that the DTAH modification effectively enhances the network’s capability to recognize varied disease patterns without requiring larger backbone networks.

**Table 4:** Results of other advanced algorithms.

Method	mAP50 (%)	Params (M)	FPS
RetinaNet R-50	70.34	36	65
Faster R-CNN R-101	72.05	44	44
YOLOv8+DTAH	77.3	11.1	123

## 4 Discussion

Microclimate alterations across the crop growth cycle greatly influences productivity and biotic interference in onion crop. Onion production in Maharashtra has been hampered by diseases such as Twister-anthracnose complex, Stemphylium blight and Purple blotch. The twister-anthracnose complex is a devastating disease in nurseries and bulb crops of onion, which shrink onion yield up to 80-100% (Patil et al., 2018). Purple blotch along with Stemphylium, affects the leaves and bulbs, culminating in losses up to 97% (Kareem et al., 2012). Stemphylium causes severe leaf blightening and incomplete bulb development which results in reduced bulb size alongside yield loss up to 20% (Correa et al., 2023). Sustainable technologies such as artificial intelligence, robotics, machine learning, deep learning etc., can be leveraged to address these issues effectively by deploying decision support system for timely implementation of countermeasures. Digital image processing and unmanned aerial vehicles (UAVs) have been utilized to monitor the crop and spot trends in biotic and abiotic stress response under varying scenarios including humid areas, weed growth, deficiency of vegetation, and poor harvest efficiency in onion farming.

The YOLOv8s Architecture integrated with DTAH attention mechanism is an Advanced method for object recognition that evaluates photographs at high speed compared to other deep learning models. This novel method of image analysis divides an image into several grid cells and predicts the bounding box coordinates even for small instances and class probabilities using a single neural network with better localization of diseased area on leaf. This results in a faster and more precise disease identification process by enabling a more efficient and accurate image assessment (Zhang et al., 2024). This model manifested highest performance among five analogous variants of yolo showcasing precision, recall and mAP50 values of 82%, 72.7% and 77.3% respectively. In similar context, Lin et al., 2023. Used integrated multiple attention mechanisms in TSBA-YOLO - an tea leaf disease detection model-built on base architecture of yolov5 which detected tea leaf diseases with 85.35% accuracy. In another study, YOLOv7 (YOLO-T) model build using CBS attention integrated with YOLOv7 resulted in 97.3% detection accuracy while detecting tea leaf diseases (Soeb et al., 2023). Incorporation of attention mechanisms has significantly improved precision of leaf disease detection while reducing possibility of error occurrence because of variation in disease spot size. (Li et al., 2022; Sun et al., 2023; Wang and Liu, 2024),)In present study, among the classes, Twister exhibited the highest performance (Precision: 0.88, Recall: 0.841, mAP50: 0.848), demonstrating the model’s strong ability to identify diseased plants. Similarly, PB and Anthracnose showed high classification accuracy (mAP50: 0.837 and 0.824, respectively), aligning with findings from prior studies that deep learning models perform well in distinct symptom recognition (Mohanty et al., 2016; Too et al., 2019). However, Stemphylium had the weakest performance (Precision: 0.854, Recall: 0.585, mAP50: 0.746), with significant misclassification as Purple blotch and background. This issue could stem from visual similarities among foliar diseases, as observed in prior research on plant disease classification (Ferentinos, 2018).

**Figure 12:**
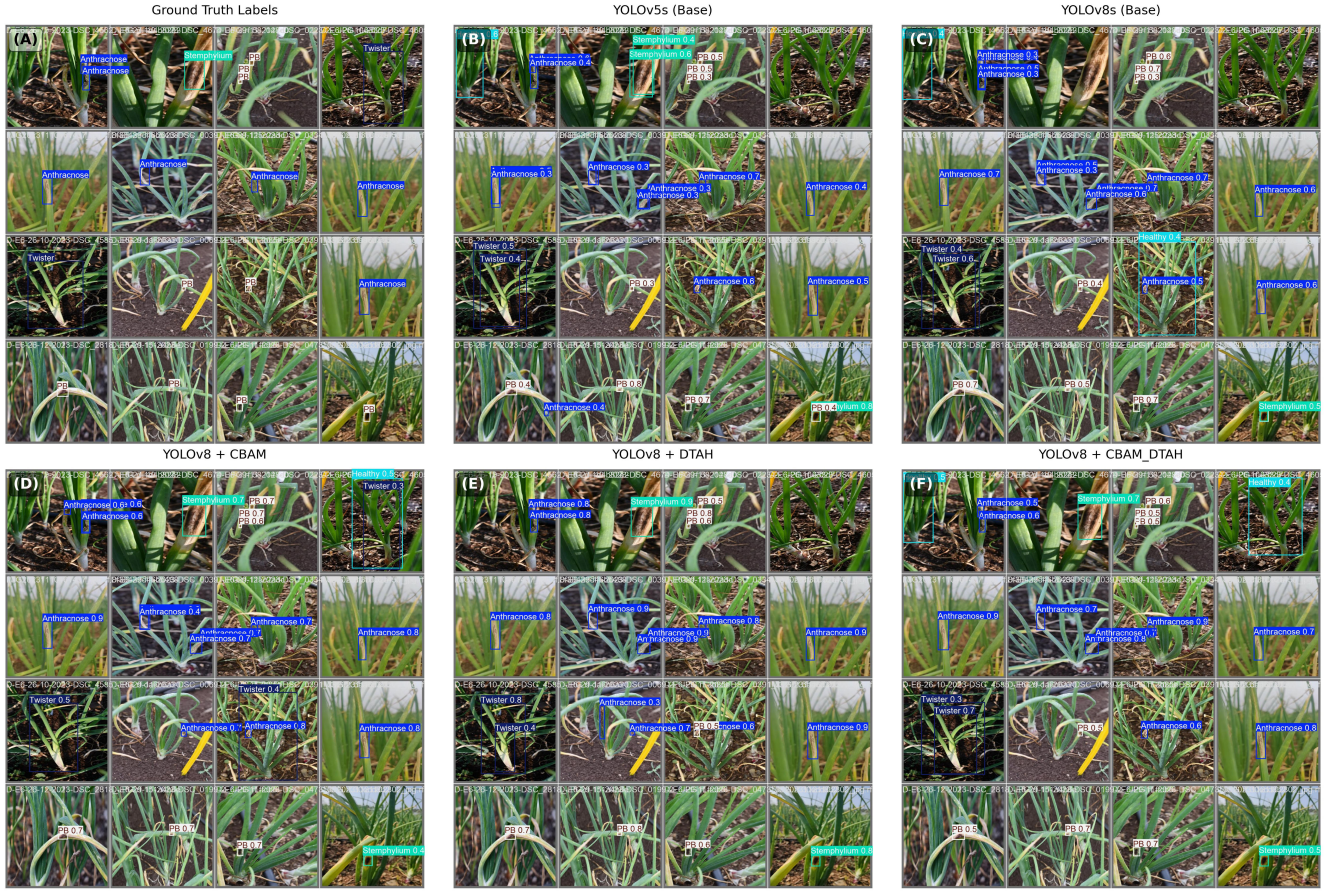
Prediction results from an object detection model.

The proposed algorithm effectively detects and identifies onion diseases by generating an optimal bounding box around the affected areas. **Figure 12**. illustrates the results of visualizing the four major categories of onion diseases, with the bounding box tightly drawn around the relevant regions. This approach ensures that the training algorithm learns features exclusively from the affected area within the bounding box, enhancing its ability to detect significantly smaller disease spots accurately. An added advantage of this method is its high image resolution, which contributes to precision. An image input size of **640 × 640 pixels** achieves the highest level of accuracy, as larger input dimensions provide more detailed information (Chen et al., 2023).

In onion disease detection, image-based approaches have primarily focused on image classification using deep learning techniques, including convolutional neural networks (CNNs) and transfer learning models (references). For example, a recent study by Jahan M. et al. (2024) explored the use of various pre-trained CNN architectures, including VGG16, VGG19, ResNet50, InceptionV3, InceptionResNetV2, and MobileNetV2, for classifying leaf diseases. Their approach involved extracting hierarchical image features using convolutional and max-pooling operations, followed by classification layers. The study also incorporated soft attention and LSTM layers to improve feature selection and sequential learning.

The primary focus of this study, however, was on the development of an onion disease object detection model for mobile camera-based applications aimed at farmers. The proposed YOLOv8-based model (YOLOv8s+DTAH) can be seamlessly integrated into both Android and iOS platforms using cross-platform frameworks like TensorFlow Lite or PyTorch Mobile. These frameworks enable efficient deployment of deep learning models on mobile devices. Similar applications have already been developed for disease detection in strawberries (Van Tran et al., 2024) and tomatoes (Zeng et al., 2023) by leveraging model quantization.

In a related study, Chen et al. (2023) proposed a system that deployed a CNN-based object detection model on mobile devices using the Keras platform. This system successfully identified and localized three types of scale pests in images. Our team has already deployed YOLOv5 within the iSARATHI mobile application for onion disease detection, providing farmers with actionable disease management advisories. The YOLOv8 model in this study builds upon these advancements, forming a key component of a digital decision support system.

Beyond disease detection, deep learning (DL) and machine learning (ML) are also being applied to other areas of precision agriculture, such as weed control, soil health management, robotic harvesting, and weather analysis. The growing use of DL in agriculture has gained traction among researchers, with notable applications including YOLOv7 for weed detection using Unmanned Aerial Vehicle (UAV)-collected data (Gallo et al., 2023) and YOLOv8n and Mask R-CNN models for automatic identification and digital phenotyping in rapeseed (Wang et al., 2023). The proposed onion disease detection model offers farmers a valuable tool for identifying affected areas, enabling more efficient disease control and better crop management.

## 5 Conclusion and future perspective

It is essential to monitor and manage the diseases at the right time and place for yield optimization in onion crop. A smart disease detection and identification system could facilitate trimming the yield gap, in addition to boosting the affluent lifestyle of farmers and extension workers. this study delivers YOLO-ODD an improved YOLOv8s model deployable into a smartphone app and detecting various onion diseases could be a digital guide for farmers. This model is able to distinguish between healthy and diseased onion classes and automatically detects four different kinds of diseases in onions. The approach works with real-world applications and Internet of Things (IoT) devices. The suggested algorithm can be used in a mobile application to help farmers getting help for their crops whenever they need it. This framework is adaptable to other plants and can be significantly adjusted to cater for additional crop diseases. More study initiatives could concentrate on gathering data on temperature and humidity, pathogenic inoculum, soil, and environmental parameters via various sensors, merging data from multiple sources, and developing an early warning model for onion plant diseases.

## Data Availability

The raw data supporting the conclusions of this article will be made available by the authors, without undue reservation.
