# Supplementary material for: YOLO-ODD: an improved YOLOv8s model for onion foliar disease detection
Source: Front Plant Sci. 2025 May 22;16:1551794. doi: 10.3389/fpls.2025.1551794 (PMC12137250; doi:10.3389/fpls.2025.1551794)

**Figure S1. Overall Architecture of Yolov8 and combine attention mechanism of model.**


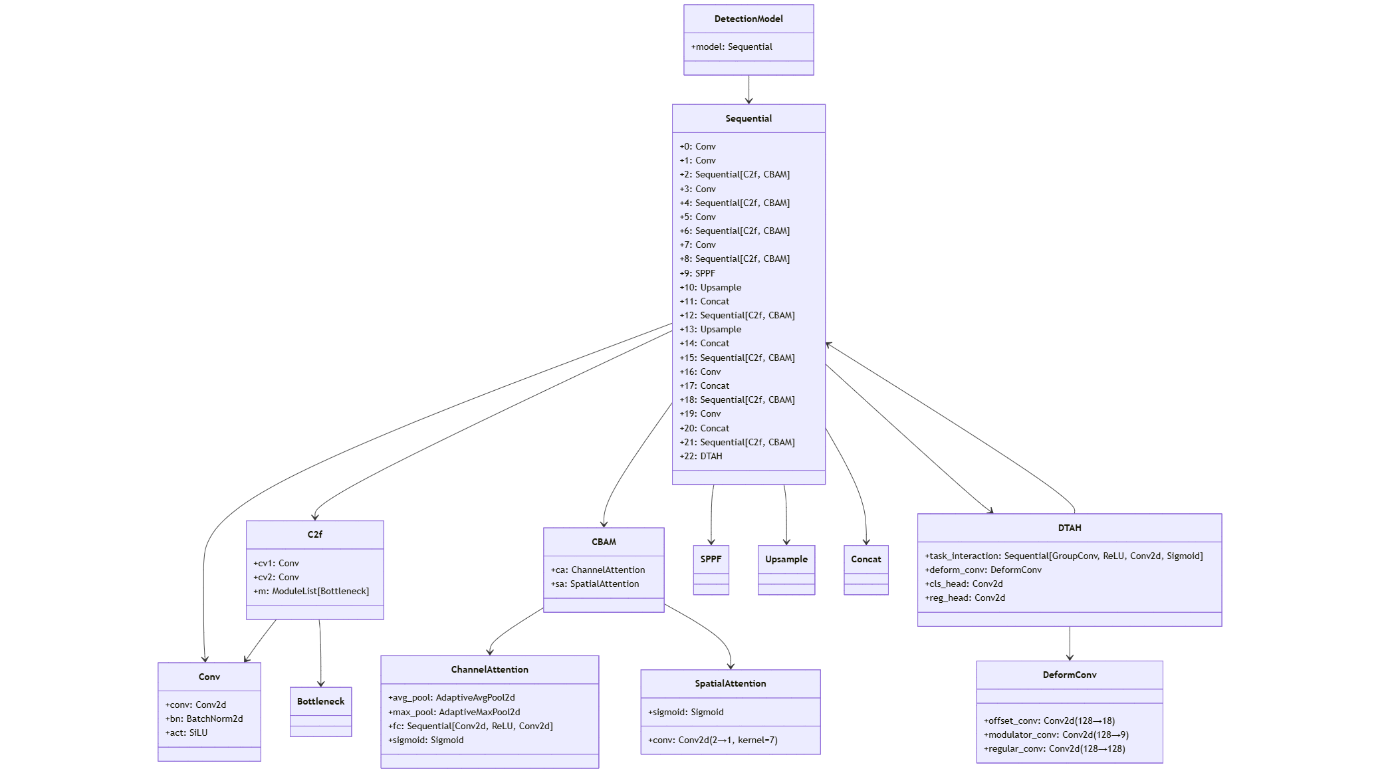


**Figure S2 Correlogram of custom dataset**


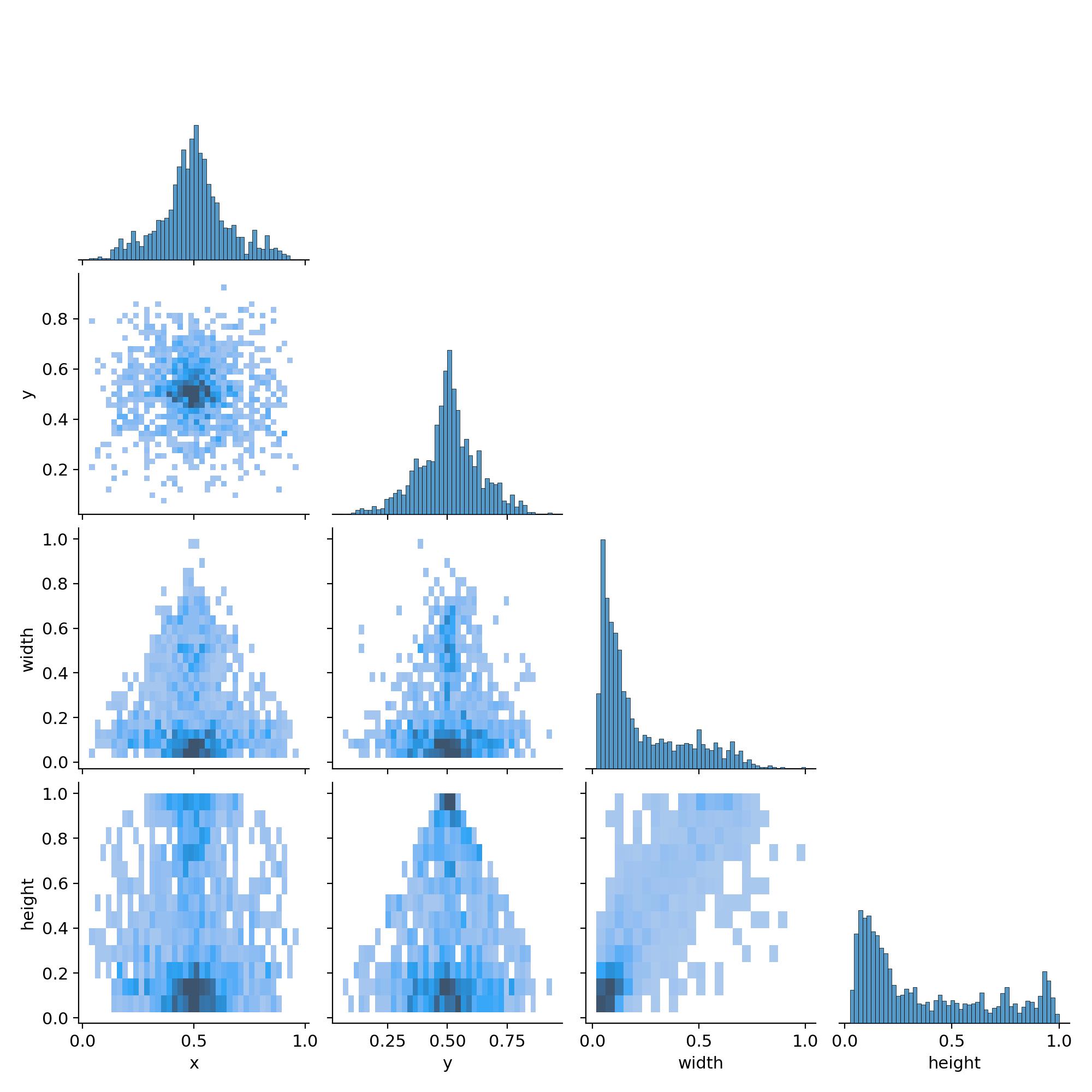


Figure S3. Precision-Recall Curve comparison across model.


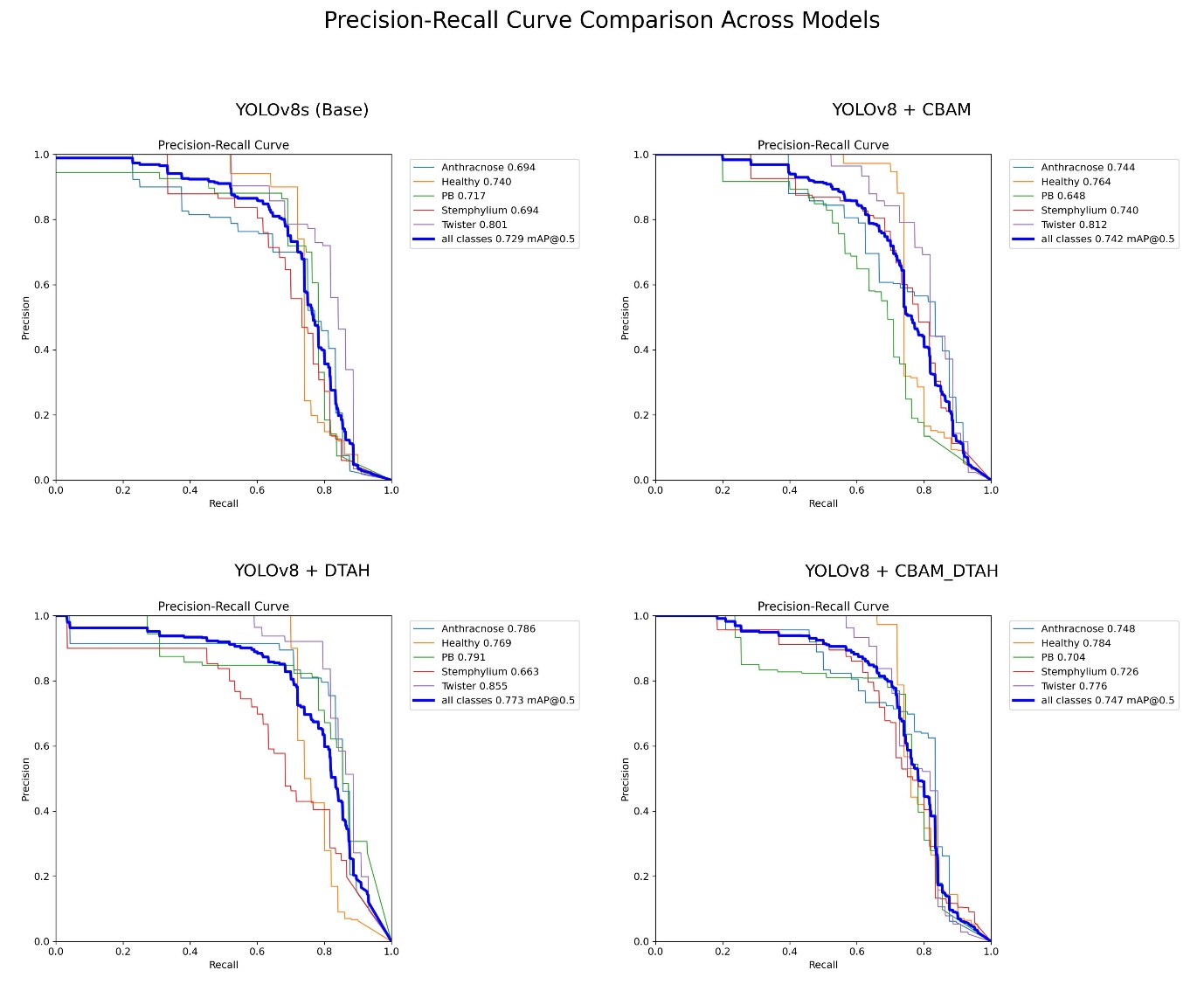

Supplement: Supplementary file 1 [file DataSheet1.docx]
